# Hantavirus in Dentistry: Occupational Exposure Considerations and a Proposed Tiered Infection Prevention and Control Framework—A Narrative Review

**DOI:** 10.1002/cre2.70481

**Published:** 2026-10-06

**Authors:** Parsa Vafaei, Morteza Banakar, Mostafa Alam, Mohammad Javad Askari, Mohsen Golkar

**Affiliations:** ^1^ Faculty of Dentistry Azad University of Isfahan (Khorasgan Branch) Isfahan Iran; ^2^ Centre of Molecular Medicine and Diagnostics (COMManD), Saveetha Dental College & Hospitals, Saveetha Institute of Medical and Technical Sciences Saveetha University Chennai India; ^3^ Department of Medical Biotechnology Shahid Sadoughi University of Medical Sciences Yazd Iran; ^4^ Department of Oral and Maxillofacial Surgery, School of Dentistry Shahid Beheshti University of Medical Sciences Tehran Iran; ^5^ Department of Prosthodontics Faculty of Dentistry, Isfahan University of Medical Sciences Isfahan Iran

**Keywords:** aerosols, Andes virus, dentistry, hantavirus, infection control, occupational exposures

## Abstract

**Objective:**

The aim of this study is to synthesize evidence on hantavirus transmission and occupational exposure pathways relevant to dentistry, and to propose an evidence‐informed tiered infection prevention and control (IPC) framework that distinguishes established from theoretical risk.

**Material and Methods:**

The initial narrative search covered database inception through May 15, 2026 in MEDLINE/PubMed, Embase, Web of Science, Scopus, Google Scholar, and WHO/CDC/ECDC/OSHA sources, followed by a targeted PubMed/MEDLINE and public‐health guidance update through August 18, 2026. Approximately 1450 records were identified. After duplicate removal, 1070 records were subjected to title/abstract screening, 215 full‐text records were assessed for eligibility, and 45 sources were included in the final synthesis, comprising 34 peer‐reviewed publications and 11 gray‐literature or public‐health guidance sources.

**Results:**

Environmental exposure to infected‐rodent contamination is the established occupational pathway. Limited person‐to‐person transmission is documented for the Andes virus (ANDV), but no dental transmission has been reported. ANDV RNA and culture‐based infectivity have been demonstrated in some oral/respiratory fluids; however, dental‐aerosol transmission has not been shown. Dental‐specific occupational risk therefore remains unquantified.

**Conclusions:**

No dental transmission has been documented, and patient‐related risk remains uncertain. Standard precautions and rodent control are foundational; suspected or confirmed ANDV warrants transmission‐based precautions and airborne precautions for aerosol‐generating procedures according to current guidance. The proposed dental tiers are evidence‐informed, author‐derived, and unvalidated.

**Abbreviations**
AGDPs: aerosol‐generating dental procedures
ANDV: Andes virus
BSL‐3: biosafety level 3
CDC: Centers for Disease Control and Prevention
COMManD: Centre of Molecular Medicine and Diagnostics
DHCWs: Dental Healthcare Workers
ECDC: European Centre for Disease Prevention and Control
EPA: Environmental Protection Agency
FFP2: Filtering Face Piece 2 Respirator
HCPS: Hantavirus Cardiopulmonary Syndrome
HEPA: high‐efficiency particulate air
HFRS: hemorrhagic fever with renal syndrome
HPS: hantavirus pulmonary syndrome
HTNV: Hantaan virus
HVAC: heating, ventilation, and air conditioning
HVE: high‐volume evacuation
ICTV: International Committee on Taxonomy of Viruses
IgG: immunoglobulin G
IgM: immunoglobulin M
IPC: infection prevention and control
MeSH: medical subject headings
NE: nephropathia epidemica
NIOSH: National Institute for Occupational Safety and Health
OSHA: Occupational Safety and Health Administration
PAPR: powered air‐purifying respirator
PCR: polymerase chain reaction
PUUV: Puumala Virus
RNA: ribonucleic acid
RT‐PCR: reverse transcription polymerase chain reaction
RT‐qPCR: quantitative reverse transcription polymerase chain reaction
SARS‐CoV‐2: severe acute respiratory syndrome coronavirus 2
ssRNA: single‐stranded ribonucleic acid
WHO: World Health Organization

## 1 Introduction

Zoonotic infections account for a substantial proportion of emerging infectious diseases and can create occupational concerns when animal‐borne pathogens enter healthcare settings. Hantaviruses are an important example because human infection is usually acquired from infected rodents and can cause severe hemorrhagic fever with renal syndrome (HFRS) or hantavirus cardiopulmonary syndrome (HCPS). The World Health Organization 2026b currently estimates approximately 10,000 to more than 100,000 human hantavirus infections worldwide each year. Older literature reported 150,000–200,000 annual HFRS cases, predominantly in Asia; these historical HFRS‐focused estimates derive from different surveillance periods and case definitions and should not be interpreted as directly comparable with the current all‐hantavirus estimate (Jonsson et al. 2010; World Health Organization 2026a).

The established route of human infection is inhalation of aerosolized virus from infected rodent urine, feces, or saliva, with contaminated surfaces and, less commonly, rodent bites providing additional routes (Jonsson et al. 2010; World Health Organization 2026a). Clinically important orthohantaviruses primarily cause disease through vascular endothelial dysfunction, increased permeability, thrombocytopenia, and organ‐specific pulmonary or renal injury; these features are relevant to dental triage because early fever, constitutional symptoms, thrombocytopenia, or mucosal bleeding may precede the fully developed syndrome (Vial et al. 2023). Andes Virus Outbreak Working Group 2026 is epidemiologically distinct because limited person‐to‐person transmission has been documented, typically after close or prolonged contact with symptomatic patients (Martínez et al. 2020; World Health Organization 2026c).

Dentistry involves close proximity to the oral cavity and procedures such as ultrasonic scaling, high‐speed handpiece use, and air–water syringe use that generate droplets and aerosols containing saliva and, at times, blood (Allison et al. 2024; Peng et al. 2020). This creates a biologically plausible patient‐related exposure pathway when a transmissible pathogen is present in oral or respiratory fluids. However, the COVID‐19 experience is used here only to inform general aerosol‐control principles; the transmission efficiency, aerosol stability, and infectious dose of SARS‐CoV‐2 cannot be extrapolated directly to hantaviruses. For hantavirus, the established occupational hazard remains rodent‐contaminated environments, whereas patient‐derived dental‐aerosol transmission is unconfirmed.

A dental‐practice commentary published in July 2026 emphasized awareness of hantavirus, general infection‐control measures, ventilation, rodent control, and postponement of non‐urgent treatment in suspected cases (Siddig et al. 2026). The present review extends that discussion by separating established environmental exposure from theoretical patient‐related dental exposure, distinguishing RNA detection from laboratory infectivity and proven transmission, integrating updated 2026 public‐health guidance, and proposing an explicitly author‐derived tiered IPC framework with its evidentiary limitations stated. Accordingly, the aims are (1) to summarize clinically relevant epidemiology and transmission, (2) to evaluate oral‐fluid and aerosol evidence relevant to dentistry, (3) to describe occupational exposure considerations without implying a formal quantitative risk assessment, and (4) to propose an evidence‐informed, tiered IPC framework for further validation. We present this article in accordance with the Narrative Review reporting checklist.

## 2 Materials and Methods

This narrative review evaluates hantavirus virology and epidemiology; established rodent‐to‐human transmission; person‐to‐person transmission, particularly of Andes Virus Outbreak Working Group 2026; viral RNA detection and infectivity in saliva and other oral fluids; aerosol‐generating dental procedures (AGDPs); occupational exposure; and infection prevention and control (IPC) relevant to dental services.

The initial literature search covered database inception through May 15, 2026 and included MEDLINE/PubMed, Embase, Web of Science, Scopus, and Google Scholar. The database search was supplemented by authoritative gray‐literature and public‐health sources from the World Health Organization 2026b Centers for Disease Control and Prevention (CDC), the European Centre for Disease Prevention and Control 2024, and Occupational Safety and Health Administration 2021, as well as backward reference searching of relevant publications. A targeted update of PubMed/MEDLINE and authoritative WHO, CDC, and ECDC sources was conducted through 18 August 2026 to identify publications and guidance released after the original search, including the July 2026 publication addressing hantavirus in dental practice. Individual platform‐specific run dates within the initial search period were not retained; therefore, May 15, 2026 is reported as the common cutoff date for the initial search rather than assigning retrospective database‐specific dates. No language or publication‐type restriction was applied at the database‐query stage.

The PubMed/MEDLINE search strategy was as follows: (“Hantavirus”[MeSH Terms] OR hantavirus*[tiab] OR orthohantavirus*[tiab] OR “Andes virus”[tiab] OR “Puumala virus”[tiab] OR “Hantaan virus”[tiab] OR “Seoul virus”[tiab] OR “Sin Nombre virus”[tiab]) AND (“Dentistry”[MeSH Terms] OR dent*[tiab] OR “Saliva”[MeSH Terms] OR saliva*[tiab] OR “oral fluid*”[tiab] OR “gingival crevicular fluid”[tiab] OR aerosol*[tiab] OR bioaerosol*[tiab] OR “Occupational Exposure”[MeSH Terms] OR “occupational exposure*”[tiab] OR “healthcare worker*”[tiab] OR “health care worker*”[tiab] OR “Infection Control”[MeSH Terms] OR “infection control”[tiab] OR “infection prevention”[tiab]). Equivalent combinations of controlled vocabulary and free‐text terms were adapted to the syntax and indexing structure of the other databases.

Eligible evidence comprised original human studies, laboratory or animal studies with direct implications for human transmission or infectivity, relevant evidence syntheses, and authoritative public‐ or occupational‐health guidance. Purely veterinary studies without relevance to human exposure, publications unrelated to healthcare or dental exposure, and opinion‐based publications without supporting evidence were excluded.

Approximately 1450 records were identified across the bibliographic and gray‐literature searches. Approximately 380 duplicate records were removed, leaving 1070 records for title/abstract screening. Of these, 855 records did not proceed to full‐text assessment. The full texts of 215 records were assessed against the stated eligibility criteria, of which 170 were not retained. The final evidence synthesis therefore comprised 45 sources: 34 peer‐reviewed publications and 11 gray‐literature or public‐health guidance sources. A reason‐specific numerical breakdown of the 170 full‐text exclusions was not prospectively recorded; therefore, exclusion categories are described according to the eligibility criteria rather than assigning retrospective counts to individual exclusion reasons. The same study‐flow numbers are reported consistently in the abstract, methods, and literature selection.

Initial title/abstract and full‐text relevance screening was performed by the lead reviewer and was not independently duplicated. Key transmission, oral‐fluid, occupational‐exposure, and IPC sources were subsequently cross‐checked by coauthors during evidence synthesis. Because independent duplicate screening was not performed, calculation of an inter‐rater agreement statistic such as Cohen's kappa was not applicable. This single‐reviewer screening process, together with the absence of prospectively recorded reason‐specific exclusion counts, represents a methodological limitation of the review. Selection bias toward studies reporting positive oral‐fluid findings and evidence from ANDV‐endemic settings also remains possible.

## 3 Hantavirus: Taxonomy, Virology, and Biological Properties

For dental risk interpretation, taxonomy is relevant because human disease is caused by mammalian orthohantaviruses rather than by all members of the Hantaviridae family. Current ICTV classification places the family Hantaviridae in the class Bunyaviricetes and order Elliovirales; the human‐pathogenic rodent‐borne viruses discussed in this review belong principally to the subfamily Mammantavirinae, genus Orthohantavirus (Bradfute et al. 2024).

### 3.1 Taxonomy, Genome Organization, and Key Pathogenic Species

Under current ICTV taxonomy, Hantaviridae contains several subfamilies and genera, whereas clinically important rodent‐borne human pathogens such as Andes, Sin Nombre, Hantaan, Seoul, Puumala, and Dobrava viruses are orthohantaviruses within the subfamily Mammantavirinae (Bradfute et al. 2024). The commonly used “Old World” and “New World” groupings are clinical–geographic descriptors rather than formal taxonomic ranks: HFRS is associated mainly with Puumala, Hantaan, Seoul, and Dobrava viruses in Europe and Asia, whereas HCPS/HPS is associated with viruses, including Sin Nombre and Andes in the Americas. Ortho‐hantaviruses are enveloped, pleomorphic viruses with a tri‐segmented, negative‐sense single‐stranded RNA genome encoding the nucleocapsid protein (S segment), glycoprotein precursor (M segment), and RNA‐dependent RNA polymerase (L segment). ANDV remains the only hantavirus for which person‐to‐person transmission is well documented (Bradfute et al. 2024; World Health Organization 2026c).

### 3.2 Environmental Stability and Inactivation

Certain environmental conditions (e.g., cool, low‐humidity conditions) allow hantaviruses to remain infectious for long periods of time, even weeks, in dry rodent excreta. It is known that hantaviruses are very sensitive to disinfection, especially with UV light, bleach, alcohol (≥ 70%), and formaldehyde. These characteristics offer guidance to surface disinfection and to the control of aerosols and the airflow in the healthcare and dental environments (Plyusnin et al. 1996).

### 3.3 Global Epidemiology and Geographic Distribution

WHO currently estimates approximately 10,000 to more than 100,000 hantavirus infections worldwide each year, with the greatest burden in Asia and Europe (World Health Organization 2026a). Older reviews reported approximately 150,000–200,000 HFRS cases annually, largely from Asia (Bi et al. 2002; Jonsson et al. 2010). These figures should not be combined: the older estimate is HFRS‐specific and reflects earlier surveillance periods, whereas the current WHO figure is a broader contemporary estimate of hantavirus infections across syndromic categories.

### 3.4 Worldwide Burden and Distribution

Global burden estimates vary according to case definition, diagnostic access, surveillance intensity, and underreporting. Current WHO material describes approximately 10,000 to more than 100,000 infections annually worldwide, with many thousands of HFRS cases in East Asia and Europe and substantially fewer HCPS cases in the Americas (World Health Organization 2026a). Historical HFRS‐only estimates of 150,000–200,000 cases per year should be interpreted in their original epidemiological context rather than as an additive or directly comparable global total (Jonsson et al. 2010).

### 3.5 Emerging Hotspots and Implications for Dental Settings

The ecological and epidemiological patterns of hantavirus disease are strongly influenced by reservoir ecology, climate, and human exposure to rodent habitats (Tian and Stenseth 2019). Travel can also result in case detection outside endemic areas. The 2026 M/V Hondius ANDV outbreak illustrates this possibility: as of July 2, 2026, WHO reported 13 cases (12 laboratory‐confirmed and one probable), including three deaths. Epidemiological and genomic evidence supported limited human‐to‐human transmission aboard the ship, and all identified contacts completed 42 days of follow‐up without additional secondary cases, after which WHO considered the outbreak contained (World Health Organization 2026c).

Healthcare employees in endemic areas have significant background risks. Endemic areas may include Scandinavia for the Puumala virus, East Asia for the Hantaan virus, and the Southern Cone of South America for the Andes virus. In non‐endemic areas, a thorough travel history, such as recent cruises, ecotourism, or travel to South America, is essential for patient triage. The 2026 MV Hondius outbreak underscores the need for precaution when a patient is experiencing febrile prodromal symptoms and is known to have relevant exposures (Vaheri et al. 2013; World Health Organization 2026a). Assessment of dental risk is heavily dependent on regional hantavirus ecology, the patient's residence, and their travel history. Figure 1 depicts the global distribution of important clinically relevant hantaviruses and their dental triage implications.

**Figure 1:**
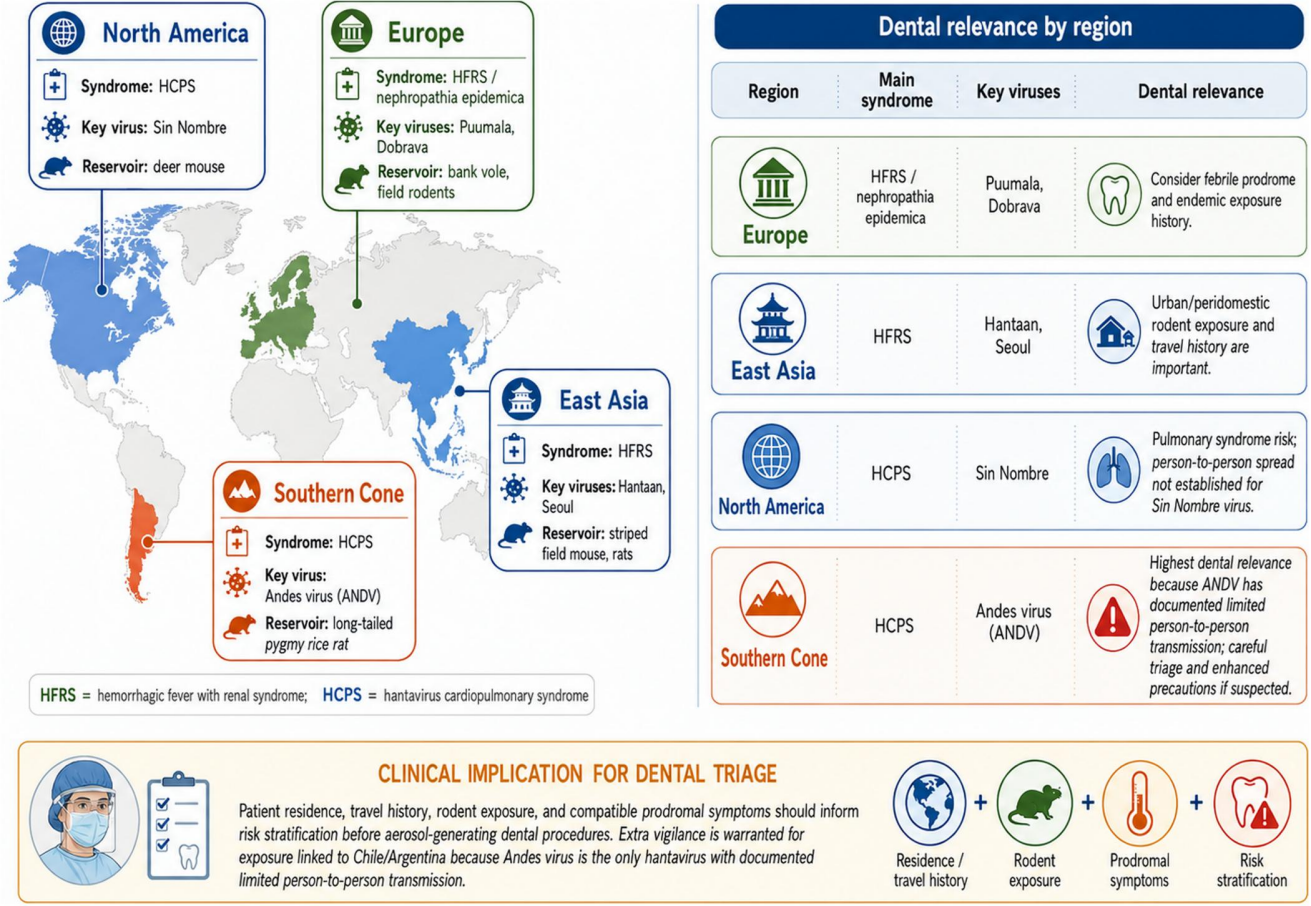
Geographic distribution of clinically relevant hantaviruses and implications for dental risk stratification. The figure summarizes the major regional patterns of clinically relevant hantaviruses, their principal syndromic presentations, representative viral species, reservoir hosts, and implications for dental triage. Europe is mainly associated with HFRS/nephropathia epidemica caused by Puumala and Dobrava viruses; East Asia with HFRS caused by Hantaan and Seoul viruses; North America with HCPS caused by the Sin Nombre virus; and the Southern Cone of South America with HCPS caused by the Andes virus. Dental triage should integrate residence or travel history, potential rodent exposure, compatible symptoms, and procedure urgency. Heightened patient‐related vigilance is principally relevant to ANDV because limited person‐to‐person transmission is documented. ANDV, Andes virus; HCPS, HCPS; and HFRS, HFRS. *Note:* This figure is a conceptual triage aid. It does not demonstrate or imply proven transmission during dental care or through dental aerosols.

## 4 Hantavirus Ecology, Reservoir Hosts, and Transmission to Humans

Hantaviruses maintain themselves in specific rodent reservoir hosts by having persistent chronic asymptomatic infections. An understanding of their ecology and how these viruses are capable of causing disease is necessary to assess the exposure risks that may occur in healthcare, including the dental profession (Jonsson et al. 2010).

### 4.1 Reservoir Hosts and Zoonotic Transmission

Each hantavirus has a dominant reservoir and has the capacity to cause chronic infection without disease manifestations. Examples of these hosts include the deer mouse (Peromyscus maniculatus) for the Sin Nombre virus, bank voles (Myodes glareolus) for the Puumala virus, Apodemus agrarius for the Hantaan virus, and the Andes virus (Oligoryzomys longicaudatus) (Jonsson et al. 2010). This coevolution permits the continued, chronic, asymptomatic shedding of the infectious virus in host secretions.

The major means by which humans contract zoonoses is through the inhalation of aerosolized viruses that have been shed from rodents in excreta or saliva. Other zoonotic transmission routes include contact with fomites that have touched the skin or mucus membranes, and less frequently, rodent bites (Centers for Disease Control and Prevention n.d.; Jonsson et al. 2010). Human infection typically includes the disturbance of rodent habitats, whether that be cleaning in confined spaces, farmwork, or peridomestic exposure. The spillover risk of infection is greatly impacted by the environment and climate, which influence the population dynamics of rodents (Centers for Disease Control and Prevention n.d.; Tian and Stenseth 2019).

### 4.2 Implications for Dental Settings

For dental facilities, the established hantavirus hazard is environmental exposure to infected rodents or rodent‐contaminated material in clinic‐adjacent spaces, storage areas, basements, waste areas, or other locations where rodent infestation can occur. Rodent exclusion, safe cleanup of contaminated areas, and facility hygiene are therefore core prevention measures (Centers for Disease Control and Prevention n.d.; World Health Organization 2026a). Patient‐related exposure is a separate and less certain pathway. It is biologically plausible mainly for ANDV because limited person‐to‐person transmission and infectious virus in some human body‐fluid samples have been documented; however, transmission during dental treatment or from dental aerosols has not been demonstrated. Thus, environmental rodent exposure is established, whereas patient‐derived dental transmission remains theoretical.

## 5 Clinical Manifestations Relevant to Healthcare

Hantavirus infections result in varying degrees of clinical syndromes with regard to the geographic clade of the virus. Understanding the clinical course of these syndromes is essential for dental practitioners to allow for the identification of patients who may present in the early stages of the infectious process (Vial et al. 2023).

### 5.1 HFRS and HCPS Clinical Course

HFRS includes five phases: a febrile phase lasting 3–7 days with associated headache, myalgia, and abdominal pain, and subsequent phases of hemorrhagic, oliguric, diuretic, and convalescent. The classic triad for this syndrome is fever, hemorrhage, and renal failure (Mustonen and Vaheri 2013; Vial et al. 2023). In contrast, HCPS is a biphasic disease with an initial 3‐ to 5‐day prodromal period of fever, myalgia, headache, and some gastrointestinal symptoms, followed by a sudden severe impact of the disease on the cardiopulmonary system with cough, dyspnea, and bilateral pulmonary infiltrates. Patients then progress to hypoxia and cardiogenic shock (Hjelle and Torres‐Pérez 2010; Vial et al. 2023).

If a patient‐related exposure were to occur in dentistry, an unrecognized prodromal or early symptomatic ANDV infection would be of greatest theoretical concern because person‐to‐person transmission has been associated mainly with close or prolonged contact during symptomatic illness (Martínez et al. 2020; Centers for Disease Control and Prevention 2026). Nevertheless, no study has demonstrated transmission during dental care, and the dental‐specific magnitude of this risk cannot currently be quantified.

### 5.2 Diagnosis and Management

Diagnosis is based on serological detection of IgM and IgG antibodies for the specific virus and RT‐PCR (preferably in the acute stage) (Vial et al. 2023; World Health Organization 2026a). Samples of suspected cases must be handled as biohazards. At present, no specific treatment exists, and care is fully supportive, including the management of fluids and electrolytes, support of the respiratory system, and renal replacement therapy when necessary. Transferring patients to an intensive care unit is essential to improve outcomes, especially in HCPS (Vial et al. 2023).

## 6 Human‐to‐Human Transmission: Evidence and Implications

Person‐to‐person transmission is not a general property of all hantaviruses. It is well documented for Andes Virus Outbreak Working Group 2026, although routine human‐to‐human transmission has not been established for the other major human‐pathogenic orthohantaviruses discussed in this review (Toledo et al. 2022; World Health Organization 2026c). This species‐specific distinction is central to infection‐control interpretation in healthcare and dentistry.

### 6.1 Human‐to‐Human Transmission of Hantavirus: Evidence

A systematic review and multiple outbreak investigations support person‐to‐person transmission of ANDV, including household, intimate contact, social cluster, and occasional healthcare‐associated transmission (Martínez et al. 2020; Toledo et al. 2022). The 2018–2019 Chubut outbreak in Argentina demonstrated substantial clustering and super‐spreading, whereas earlier investigations documented nosocomial transmission in a small number of healthcare workers. Close and prolonged contact, including exposure to saliva or respiratory secretions, has been implicated epidemiologically, but the exact relative contribution of droplets, aerosols, direct mucosal contact, and contaminated surfaces remains incompletely defined. For Sin Nombre, Puumala, Hantaan, and other major hantaviruses, healthcare serosurveys and epidemiological investigations have generally not demonstrated comparable person‐to‐person transmission.

### 6.2 Dental Setting Concerns

In dental settings, suspected or confirmed ANDV infection warrants greater caution than other hantavirus infections because person‐to‐person transmission is documented. This does not mean that dental procedures are a proven transmission route. No dental transmission event has been reported, and no study has shown that AGDPs produce infectious ANDV aerosols. Dental triage should therefore combine compatible symptoms with epidemiological risk, including rodent exposure, residence or travel in endemic areas, or close contact with a symptomatic suspected or confirmed ANDV case, and should follow current public‐health guidance for suspected infection (Centers for Disease Control and Prevention 2026; World Health Organization 2026a).

## 7 Hantavirus in Saliva and Oral Fluids: Evidence and Dental Relevance

Hantavirus findings in saliva and other oral fluids require separation of three different levels of evidence: (1) detection of viral RNA or antigen, (2) demonstration of replication‐competent virus in laboratory infectivity assays, and (3) epidemiologically proven transmission by a specific oral or dental route. Viral RNA detection alone does not demonstrate infectivity or transmission. However, the evidence has advanced beyond RNA detection alone for ANDV: a 2024 prospective study recovered infectious virus in Vero E6 culture from a subset of RT‐qPCR‐positive non‐blood fluids that included gingival crevicular fluid, saliva, nasopharyngeal specimens, and urine (Ferrés et al. 2024). This establishes biological infectivity in some clinical‐fluid samples but does not establish saliva as the route of person‐to‐person transmission and does not demonstrate dental‐aerosol transmission.

### 7.1 Detection of Hantavirus RNA and Antigen in Oral Fluids

Pettersson et al. detected Puumala virus (PUUV) RNA in saliva from 10 of 14 patients (71%) with nephropathia epidemica, with reported salivary loads of 1530–121,323 copies/mL and detection during the early symptomatic phase for up to 9 days after symptom onset (Pettersson et al. 2008). In the larger prospective ANDV study of 131 confirmed cases, ANDV RNA was detected in saliva in 12% of acute‐phase samples and 11% of convalescent samples, whereas gingival crevicular fluid was RT‐qPCR‐positive in 30% of acute‐phase samples. Importantly, among RT‐qPCR‐positive non‐blood fluids tested during acute illness, including gingival crevicular fluid, saliva, nasopharyngeal swabs, and urine, infectivity assays were positive in 18 of 43 samples (42%) overall; the publication did not provide a saliva‐specific infectivity proportion. Laboratory infectivity therefore should not be conflated with a measured transmission rate (Ferrés et al. 2024). Immunohistochemical studies have also demonstrated ANDV antigen in salivary‐gland secretory cells (Pizarro et al. 2020). A summary of evidence for hantavirus RNA, antigen, and infectivity in human saliva and oral fluids is provided in Table 1.

**Table 1:** Summary of evidence for hantavirus RNA, antigen, and infectivity in human saliva and oral fluids.

Study	Virus/population	Oral‐fluid detection	Laboratory infectivity	Timing/interpretation	Reference
Pettersson et al. (2008)	PUUV; 14 hospitalized nephropathia epidemica patients	Saliva RT‐qPCR‐positive in 10/14 (71%); 1,530‐121,323 copies/mL	Not assessed in this study	Early symptomatic phase to day 9; RNA detection alone does not establish infectivity.	(Pettersson et al. (2008))
Ferrés et al. (2024)	ANDV; prospective cohort of 131 confirmed cases	Saliva RNA: 12% acute and 11% convalescent; gingival crevicular fluid: 30% acute	18/43 (42%) aggregate RT‐qPCR‐positive acute non‐blood fluids (GCF, saliva, NPS, and urine) were positive in Vero E6 assays; no saliva‐specific rate was reported.	Acute/convalescent phases. Culture infectivity in some fluids does not prove saliva‐mediated or dental transmission.	(Ferrés et al. (2024))
Pizarro et al. (2020)	ANDV; salivary‐gland tissue from fatal cases	ANDV antigen detected by immunohistochemistry in salivary‐gland secretory cells	Not assessed	Supports a potential salivary shedding source; does not establish infectious saliva or transmission.	(Pizarro et al. (2020))
Hardestam et al. (2009)	PUUV/HTNV/ANDV; in vitro and animal experiments	Experimental studies of saliva and salivary components	Mucin showed partial antiviral activity; PUUV RNA‐positive saliva did not cause bank‐vole seroconversion.	Infectivity varies by virus, specimen, and assay; RNA positivity should not be equated with infectiousness.	(Hardestam et al. (2009))

Abbreviations: ANDV, Andes virus; HTNV, Hantaan virus; IHC, immunohistochemistry; mL, milliliter; N/A, not applicable; NE, nephropathia epidemica; PUUV, Puumala virus; RNA, ribonucleic acid; and RT‐qPCR, quantitative reverse transcription polymerase chain reaction.

### 7.2 Laboratory Infectivity and Antiviral Properties of Saliva

Earlier experimental work showed that components of human saliva can inhibit hantavirus infectivity to varying degrees. Mucin reduced Hantaan virus infectivity in vitro, whereas several other salivary proteins showed little or no inhibitory effect; PUUV RNA‐positive saliva did not produce seroconversion after inoculation into bank voles (Hardestam et al. 2009; Hardestam et al. 2009). Those studies supported caution in interpreting RNA‐positive saliva as infectious. The later ANDV study by Ferrés et al. (2024), however, demonstrated Vero E6 infectivity in a subset of RT‐qPCR‐positive non‐blood clinical fluids that included saliva and gingival crevicular fluid. The two bodies of evidence are not contradictory: infectivity differs by virus, specimen, timing, and assay, and laboratory infectivity still does not by itself prove a specific transmission route.

### 7.3 Implications for Aerosol‐Generating Dental Procedures

The oral‐fluid evidence supports biological plausibility for exposure during dental care, particularly for ANDV, but does not permit a dental transmission probability to be estimated. No study has demonstrated that AGDP‐generated aerosols from an ANDV‐infected patient contain a replication‐competent virus, and no dental transmission event has been reported. Accordingly, dental occupational risk should be described as uncertain rather than “minimal” or “low” on the basis of absent dental cases alone. Where a patient has suspected or confirmed ANDV infection, precautionary escalation should be based on current healthcare IPC guidance and the aerosol‐generating nature of the planned procedure, not on an assumption that salivary or dental‐aerosol transmission has been proven (Centers for Disease Control and Prevention 2026; Ferrés et al. 2024; World Health Organization 2026a).

## 8 Aerosol‐Generating Dental Procedures and Mitigation Strategies

Dental procedures generate droplets and aerosols, but most quantitative mitigation studies use bacterial tracers, fluorescent particles, bacteriophages, or respiratory viruses other than hantavirus (Allison et al. 2024; Harrel and Molinari 2004). Evidence developed during the COVID‐19 pandemic is therefore used only to understand aerosol physics and source‐control options, not to infer that hantavirus has SARS‐CoV‐2‐like transmissibility. Differences in the viral structure, environmental stability, infectious dose, shedding, and route of transmission preclude direct extrapolation. The evidence in this section should be considered indirect engineering evidence for a theoretical hantavirus exposure model.

### 8.1 Bioaerosol Characteristics and Spatial Dispersion in Dental Settings

AGDPs involve the use of high‐speed handpieces, ultrasonic scalers, air–water syringes, and surgical drills, and create a mixture of droplets and aerosols of a wide size range (Allison et al. 2024; Harrel and Molinari 2004). Particles with a diameter of less than 5 µm can remain airborne for tens of minutes (or longer) in poorly ventilated operatories and can travel more than 1.5 m, whereas droplets with sizes in the range of 5–50 µm sediment more rapidly (Harrel and Molinari 2004; Zemouri et al. 2017). Most of the data regarding the size and sedimentation characteristics of droplets and aerosols are based on studies of tracers (bacteria and SARS‐CoV‐2) in standard dental operatories that are ventilated with an average of 6–12 air changes per hour (Allison et al. 2024; Zhu et al. 2022). Dental bioaerosols are composed primarily of the microbial flora in the patient's mouth, and the bioaerosols can be contaminated with viruses if the patient is in a viral (e.g., viremic) phase (Allison et al. 2024; Zemouri et al. 2017).

### 8.2 Efficacy of Aerosol Mitigation Strategies

High‐volume evacuation (HVE) is an important dental source‐control measure, but its performance depends on suction flow, tip design and position, procedure, and room conditions. The previously reported value of 2.5–3.0 L/min was not a valid HVE flow rate and has been removed. In a dental HVE evaluation, measured clinic HVE flow rates were approximately 104–194 L/min, and experimental testing used 100 and 200 L/min; these values illustrate the order of magnitude rather than a universal standard (Onuche 2021). Because reported percentage reductions vary substantially across study designs, the earlier distance‐specific reduction percentages are also omitted here. Rubber dam isolation when feasible, four‐handed dentistry, and appropriate local or extra‐oral evacuation may further reduce procedure‐generated contamination, and adequate ventilation aids removal of airborne particles (Allison et al. 2024; Zhu et al. 2022).

### 8.3 Applicability to Hantavirus and Constraints

There are no dental studies directly measuring the aerosol stability, infectious dose, or transmission efficiency of hantaviruses. Therefore, even where general aerosol‐control measures reduce particles, their effectiveness specifically against infectious hantavirus generated during dental treatment is unknown. Surrogate models and appropriately contained laboratory studies will be required before hantavirus‐specific quantitative mitigation claims can be made.

The evidence therefore supports two qualitatively different exposure concepts: an established environmental/rodent route and a theoretical patient/dental route. The latter is biologically plausible for ANDV because infectious virus has been recovered from some non‐blood clinical fluids, but it remains unvalidated for dental aerosols. Figure 2 is intended to illustrate hypotheses for risk recognition, not to depict proven dental transmission pathways.

**Figure 2:**
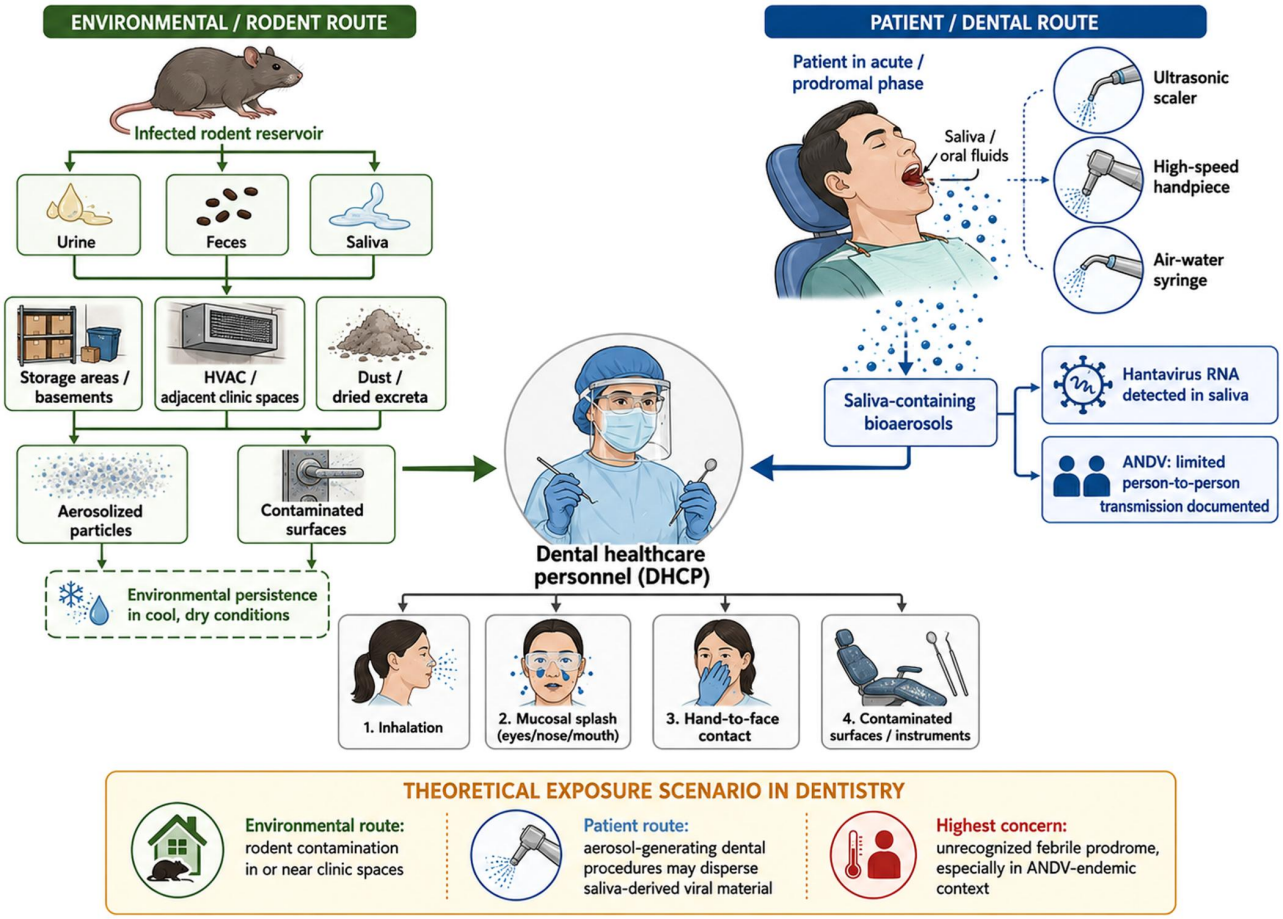
Potential hantavirus exposure pathways relevant to dental healthcare settings. The figure illustrates two conceptually distinct routes. The environmental/rodent route is established and involves contaminated clinic‐adjacent spaces, storage areas, basements, dust, dried excreta, surfaces, or other materials contaminated by infected rodents. The patient/dental route is theoretical and illustrates possible exposure to saliva‐ or oral‐fluid–containing droplets or aerosols during procedures in a patient with acute ANDV infection. Potential occupational contacts include inhalation, mucosal splash, hand‐to‐face transfer, and contaminated surfaces or instruments. ANDV, Andes virus; DHCP, dental healthcare personnel; HVAC, heating, ventilation, and air conditioning; and RNA, ribonucleic acid. *Note:* Patient‐derived transmission during dental treatment, including dental‐aerosol transmission, has not been demonstrated. This pathway is hypothetical, and the figure is not a validated risk model.

### 8.4 Source Control and Environmental Measures

Pre‐procedural mouth rinses with chlorhexidine, povidone‐iodine, etc. significantly reduce bacterial contamination of dental aerosols (Mi‐Rim Yun et al. 2025). The use of a rubber dam, HVE, four‐handed dentistry, and extra‐oral suction, combined with environmental control measures, also limits the generation and dispersion of aerosols at the source. Controlling the dental unit waterlines and HVAC systems with HEPA filters reduces airborne particles (Allison et al. 2024).

### 8.5 Applicability to Hantavirus

Measures such as HVE, rubber dam isolation, appropriate ventilation, and personal protective equipment have a rational source‐control basis in dentistry. Their inclusion in the proposed framework is an extrapolation from general dental aerosol‐control and healthcare IPC evidence. It should not be interpreted as proof that these measures prevent hantavirus transmission during dental procedures, because dental hantavirus aerosol transmission itself has not been demonstrated (Allison et al. 2024; World Health Organization 2026a).

## 9 Occupational Exposure Considerations for Dental Healthcare Personnel

The best‐established occupational hazard for hantavirus is exposure to infected rodents or rodent‐contaminated excreta and materials. Healthcare‐associated transmission is uncommon overall, although nosocomial ANDV transmission has been documented outside dentistry. No published case of hantavirus transmission to dental healthcare personnel was identified. The absence of reported dental cases should not be interpreted as proof of a low dental risk, because dental‐specific epidemiological studies are lacking and the true risk cannot presently be quantified (Martinez‐Valdebenito et al. 2014; World Health Organization 2026a).

### 9.1 Exposure Pathways and Risk Modifiers

The primary established occupation‐acquired pathway is environmental exposure to rodent reservoirs or contaminated rodent excreta, including exposure during cleaning or entry into infested enclosed spaces (World Health Organization 2026a). In dental healthcare, this risk can arise in storage areas, basements, waste zones, or other clinic‐associated spaces with rodent activity. A second, patient‐related route is plausible but unproven in dental practice and is principally relevant to ANDV because limited person‐to‐person transmission is documented and infectious virus has been recovered from some clinical fluids. Regional epidemiology, recent travel, rodent exposure, close contact with symptomatic ANDV cases, compatible symptoms, and the need for AGDPs modify the level of precaution.

### 9.2 Evidence Base and Uncertainty in Patient‐Related Occupational Risk

Available healthcare data indicate that patient‐to‐healthcare worker transmission of hantavirus is uncommon, but not impossible for ANDV. Earlier serosurveys found no excess hantavirus antibodies among some healthcare worker cohorts caring for infected patients, whereas separate outbreak investigations have documented occasional nosocomial ANDV transmission (Castillo et al. 2000; Martinez‐Valdebenito et al. 2014). No dental‐specific transmission case was identified in this review. Consequently, the appropriate conclusion is not that dental occupational risk is definitively low, but that it is unquantified and probably context‐dependent; uncertainty is greatest for AGDPs involving a symptomatic patient with suspected or confirmed ANDV infection.

### 9.3 Infection Prevention and Control: Framework for Dental Settings

The dental framework below adapts current WHO and CDC healthcare IPC recommendations together with established dental standard precautions and aerosol source‐control principles. It is not an official WHO, CDC, or ECDC dental hantavirus protocol. Standard precautions apply to all patients; for suspected or confirmed hantavirus infection, WHO advises transmission‐based precautions and airborne precautions for aerosol‐generating procedures, whereas CDC 2026 ANDV guidance recommends an airborne infection isolation room, gown, gloves, eye protection, and an N95 or higher level respirator in healthcare settings (Centers for Disease Control and Prevention 2026; Kohn et al. 2003; World Health Organization 2026a).

### 9.4 Standard and Transmission‐Based Precautions

Standard precautions include hand hygiene, gloves as indicated, appropriate mask and eye/face protection, safe handling of blood and body fluids, instrument reprocessing, and environmental cleaning (Kohn et al. 2003). When suspected or confirmed ANDV infection cannot be deferred for urgent care, escalation should follow current transmission‐based guidance. WHO advises airborne precautions for aerosol‐generating procedures, and CDC recommends an N95 or higher level respirator, gown, gloves, eye protection, and airborne infection isolation for suspected or confirmed ANDV in healthcare settings (Centers for Disease Control and Prevention 2026; World Health Organization 2026a). Rodent exclusion and safe environmental cleaning remain essential because environmental rodent exposure is the established hantavirus route.

### 9.5 Proposed Tiered Infection Control Framework

The following tiered framework is an author‐proposed synthesis that adapts current public‐health guidance to dental decision‐making. It is intended to structure triage and precaution selection, not to represent a formally validated dental risk‐assessment tool or an official requirement. The recommendations and their evidentiary basis are summarized in Table 2.

**Table 2:** Evidence‐informed, author‐proposed tiered IPC framework for hantavirus‐related considerations in dental practice.

Tier	Target patients	Key recommendations	Evidence and rationale
Tier 1: Standard	All patients	Hand hygiene; gloves as indicated; procedure‐appropriate mask and eye/face protection; standard instrument reprocessing and surface disinfection; rodent exclusion and safe environmental cleaning.	Standard precautions are foundational for dental care (Kohn et al. 2003; WHO 2026d). Rodent control addresses the established environmental hantavirus route. This tier does not imply that dental transmission has been ruled out.
Tier 2: Enhanced (epidemiological alert)	Compatible symptoms plus relevant epidemiological exposure within the previous 42 days for ANDV. For non‐ANDV exposure, consider the broader approximately 1‐8‐week interval described by WHO.	Take a detailed exposure/travel history. Consider deferring elective AGDPs when clinical suspicion is meaningful. If care proceeds, use procedure‐appropriate PPE; consider a fit‐tested N95/FFP2 for AGDPs when warranted by risk assessment or local guidance. Use HVE and rubber dam when feasible and optimize ventilation/local source control.	Precautionary tier for epidemiological alert (Allison et al. 2024; CDC 2026a; Kohn et al. 2003; WHO 2026d, 2026e; Zhu et al. 2022). Dental‐aerosol controls are supported by indirect aerosol evidence, not hantavirus‐specific trials; salivary or dental‐aerosol transmission is not treated as proven.
Tier 3: High risk (suspected or confirmed)	Suspected or laboratory‐confirmed Andes virus (ANDV) infection. Airborne precautions in this tier are limited to suspected or confirmed ANDV, in accordance with current CDC guidance, and are not applied indiscriminately to all hantaviruses.	Defer elective/non‐urgent care and arrange medical/infectious‐disease and public‐health coordination as appropriate. If emergency care is unavoidable, minimize AGDPs and follow transmission‐based/airborne precautions: airborne infection isolation when available, gown, single gloves, eye/face protection, and a fit‐tested N95/FFP2 or higher level respirator. A PAPR may be used only as an institutional alternative when indicated by the respiratory‐protection program.	Current CDC guidance for suspected/confirmed ANDV specifies gown, gloves, eye protection, an N95 or higher level respirator, and airborne infection isolation (CDC 2026a; WHO 2026d, 2026e). Double gloving is not a routine official requirement; PAPR use is optional rather than a hantavirus‐specific mandate. This dental tier is author‐proposed and unvalidated.

Abbreviations: AGDPs, aerosol‐generating dental procedures; CDC, Centers for Disease Control and Prevention; EPA, Environmental Protection Agency; FFP2, filtering facepiece 2 respirator; HVE, high‐volume evacuation; N95, N95 filtering facepiece respirator; PAPR, powered air‐purifying respirator; RNA, ribonucleic acid; and WHO, World Health Organization.

For ANDV‐focused epidemiological screening, relevant exposures should be sought during the 42 days before symptom onset, consistent with current CDC guidance and WHO contact follow‐up used during the 2026 outbreak (Centers for Disease Control and Prevention 2026; World Health Organization 2026c). The WHO notes that hantavirus symptoms more generally may begin within approximately 1–8 weeks depending on the virus; therefore, clinicians should also consider a longer interval when epidemiology suggests a non‐ANDV hantavirus exposure (World Health Organization 2026a). Elective care refers to non‐time‐critical restorative, cosmetic, and periodontal procedures. Urgent/emergency care refers to management of pain, infection, trauma, or other time‐critical conditions, including threats to the airway.

This framework is deliberately precautionary and flexible. Its dental tiers have not been prospectively validated, and local infection‐control, infectious‐disease, and public‐health guidance should take precedence when a suspected or confirmed case is encountered.

### 9.6 Personal Protective Equipment, Patient Triage, and Management of Suspected Cases

Patient triage should assess compatible symptoms and epidemiological exposures, especially within the 42 days before symptom onset for ANDV (Centers for Disease Control and Prevention 2026). When suspected or confirmed ANDV infection is present, elective treatment should be deferred, and medical/infectious‐disease assessment should be arranged. If urgent dental treatment cannot be postponed, the WHO recommends airborne precautions for aerosol‐generating procedures, and current CDC ANDV healthcare guidance specifies airborne infection isolation, gown, gloves, eye protection, and an N95 or higher level respirator (Centers for Disease Control and Prevention 2026; World Health Organization 2026a). A PAPR may be used as an alternative higher level respirator when indicated by an institutional respiratory‐protection program; it is not a universal hantavirus requirement. Routine double gloving is not required by the cited CDC/WHO guidance and has therefore been removed from the framework. Post‐exposure management should be coordinated with occupational health and public‐health authorities according to the nature of the exposure and current local guidance. The proposed tiered screening is shown in Figure 3.

**Figure 3:**
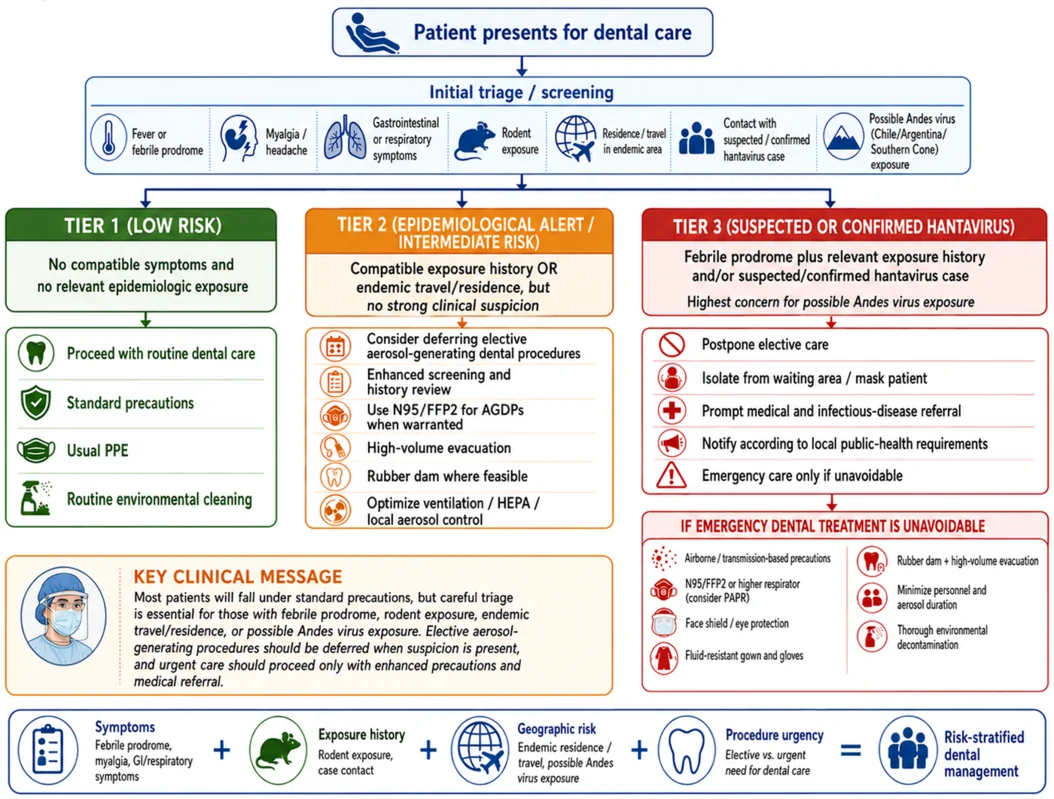
Proposed tiered screening and infection‐control pathway for suspected hantavirus risk in dental practice. This algorithm provides an author‐proposed risk‐stratified approach for dental triage. Initial assessment should consider compatible symptoms, rodent exposure, residence or travel in endemic areas, close contact with a symptomatic, suspected or confirmed hantavirus case, possible ANDV exposure, and procedure urgency. For ANDV, relevant exposure history should extend through the previous 42 days. Tier 1 supports routine care with standard precautions in the absence of a relevant alert. Tier 2 represents an epidemiological alert in which enhanced screening, possible deferral of elective AGDPs, and procedure‐appropriate source control/PPE may be considered. Tier 3 applies to suspected or confirmed ANDV infection and prioritizes postponement of elective care, medical referral, public‐health coordination, and transmission‐based/airborne precautions if urgent treatment is unavoidable. AGDPs, aerosol‐generating dental procedures; FFP2, filtering facepiece 2 respirator; HEPA, high‐efficiency particulate air; HVE, high‐volume evacuation; PAPR, powered air‐purifying respirator; and PPE, personal protective equipment. *Note:* This pathway is an evidence‐informed, author‐derived framework. The proposed dental triage and precaution pathway remains hypothetical and unvalidated. Dental‐aerosol transmission of hantavirus has not been demonstrated. Airborne precautions are specified for suspected or confirmed ANDV in accordance with current CDC guidance; national/local IPC guidance should take precedence.

### 9.7 Research Gaps, Future Directions, and Policy Implications

The most important research gap is no longer simply whether infectious ANDV can be recovered from human oral fluids; the 2024 prospective study has demonstrated infectivity in a subset of RT‐qPCR‐positive non‐blood fluids that included saliva and gingival crevicular fluid (Ferrés et al. 2024). The next priorities are to determine saliva‐ and gingival fluid–specific frequencies and titers of replication‐competent virus, define how infectivity changes across the clinical course, establish whether dental instruments can aerosolize replication‐competent ANDV from oral fluids, and quantify aerosol viability, infectious dose, and exposure–response relationships under dental conditions. Epidemiological surveillance or serological studies focused specifically on dental healthcare personnel are also needed.

These questions will require appropriately contained virological studies, dental‐aerosol models, and multicenter occupational surveillance. Surrogate‐virus experiments may help evaluate engineering controls, but results must be clearly identified as indirect evidence. Future studies should also evaluate the proposed tiered framework prospectively, including feasibility, over‐triage, resource implications, and concordance with evolving public‐health guidance. To the best of our knowledge, no study has as yet demonstrated hantavirus transmission during dental treatment or directly tested infectious hantavirus in dental bioaerosols.

Professional dental associations in Andes virus‐endemic areas, including Chile and Argentina, should develop and disseminate hantavirus‐specific infection‐control recommendations. Organizations with a global health perspective such as the WHO and CDC should discuss dental services and the potential of airborne infection in the context of dental services in their future guidance for hantavirus. There should be a recognition of hantavirus as a potential zoonotic disease in dental curricula and occupational health services in the countries with dental care services where incidences are high.

If the research gaps and the recommended policies are addressed, occupational safety will be enhanced and the ability to provide dental services in both endemic and non‐endemic countries will be greatly improved.

## 10 Conclusions

Hantavirus infection presents two distinct considerations for dentistry: an established environmental zoonotic hazard from rodent‐contaminated materials and an uncertain, species‐specific patient‐related hazard, principally involving ANDV. Person‐to‐person transmission of ANDV is documented, and viral RNA plus laboratory infectivity have been demonstrated in some oral and other non‐blood clinical fluids. However, neither transmission during dental care nor transmission through dental aerosols has been demonstrated. Dental‐specific occupational risk therefore cannot currently be quantified. Standard precautions, facility hygiene, rodent exclusion, and safe management of rodent contamination remain the foundation of prevention. For patients with suspected or confirmed ANDV infection, enhanced measures should follow current public‐health and healthcare IPC guidance, particularly airborne precautions for aerosol‐generating procedures. Consistent with current CDC recommendations, airborne isolation and an N95 or higher level respirator are specified for suspected or confirmed ANDV rather than for all hantavirus infections. The tiered framework proposed in this review is an evidence‐informed, author‐derived model intended to support precautionary dental decision‐making; it requires validation and should not replace national, local, or institutional guidance.

## Author Contributions

**Parsa Vafaei:** conceptualization, investigation, data curation, visualization, writing – original draft. **Morteza Banakar:** conceptualization, methodology, supervision, project administration, validation, writing – review and editing. (corresponding author). **Mostafa Alam:** methodology, investigation, data curation, writing – original draft. **Mohammad Javad Askari:** investigation, data curation, writing – original draft. **Mohsen Golkar:** investigation, data curation, writing – original draft. All authors have read and approved the final version of the manuscript and agree to be accountable for all aspects of the work in ensuring that questions related to the accuracy or integrity of any part of the work are appropriately investigated and resolved.

## Funding

The authors have nothing to report.

## Ethics Statement

The authors have nothing to report.

## Consent

The authors have nothing to report.

## Conflicts of Interest

The authors declare no conflicts of interest.

## Policy on Using ChatGPT and Similar AI Tools

The authors employed artificial intelligence‐based language models (Gemini and Grok) exclusively for improving the linguistic fluency, clarity, and readability of the manuscript. All scientific ideas, research design, data analysis, interpretation, and core content were conceived, developed, and written by the authors. The authors take full responsibility for the accuracy and integrity of the entire paper.

## Data Availability

Data sharing is not applicable to this article, as no new datasets were generated or analyzed during the current narrative review. All data and materials discussed in this manuscript are derived from previously published studies and publicly available sources, which are cited in the reference list. Zoonoses and Public Health (ZPH).
